# Positive Impact of Nutritional Interventions on Serum Symmetric Dimethylarginine and Creatinine Concentrations in Client-Owned Geriatric Dogs

**DOI:** 10.1371/journal.pone.0153653

**Published:** 2016-04-18

**Authors:** Jean A. Hall, Jennifer MacLeay, Maha Yerramilli, Edward Obare, Murthy Yerramilli, Heidi Schiefelbein, Inke Paetau-Robinson, Dennis E. Jewell

**Affiliations:** 1 Department of Biomedical Sciences, College of Veterinary Medicine, Oregon State University, Corvallis, Oregon, United States of America; 2 Pet Nutrition Center, Hill's Pet Nutrition, Inc, Topeka, Kansas, United States of America; 3 IDEXX Laboratories, Inc., One IDEXX Drive, Westbrook, Maine, United States of America; Hospital Universitario de La Princesa, SPAIN

## Abstract

A prospective study was conducted in client-owned geriatric dogs to evaluate the short-term effects of a test food on serum symmetric dimethylarginine (SDMA) and creatinine (Cr) concentrations. Test food contained functional lipids (fish oil), antioxidants (lipoic acid, vitamins C and E), L-carnitine, botanicals (fruits and vegetables), controlled sodium concentration, and high quality protein sources (high bioavailability and an ideal amino acid composition). Dogs (n = 210) were fed either test food or owner’s-choice foods (non-nutritionally controlled cohort). Dogs were included based on age and body weight: small (6.8 to 11.4 kg) and medium dogs (11.5 to 22.7 kg) were ≥ 9 years, whereas dogs >22.7 kg were ≥ 7 years at baseline. At baseline, all dogs had to have serum Cr concentrations within the reference interval and be free of chronic disease. Renal function biomarkers and urinalysis results at baseline, and after consuming test food or owner’s-choice foods for 3 and 6 months, were evaluated. Only dogs consuming test food showed significant decreases in serum SDMA and Cr concentrations (both *P* ≤ 0.05) across time. At baseline or during the 6-month feeding trial, 18 dogs (8.6%) had increased serum SDMA, but normal serum Cr, consistent with IRIS Stage 1 chronic kidney disease. This included 9 dogs fed test food and 9 dogs fed owner’s-choice foods. Compared with baseline, after feeding 9 dogs test food for 6 months, serum SDMA decreased in 8 dogs and increased in 1 dog. After feeding 9 dogs owner’s-choice foods for 6 months, serum SDMA decreased in 4 dogs and increased in 4 dogs (remained stable in 1 dog). The decreases in serum SDMA and Cr concentrations were significant (both *P* = 0.03) only for dogs fed test food. These results suggest that nonazotemic dogs with elevated serum SDMA (early renal insufficiency) when fed a test food designed to promote healthy aging are more likely to demonstrate improved renal function compared with dogs fed owner’s-choice foods.

## Introduction

Serum concentrations of symmetric dimethylarginine (SDMA) have been shown to detect chronic kidney disease (CKD) in cats on average 17.0 months before serum creatinine (Cr) concentration increased above the reference interval [1], and in dogs on average 9.8 months before serum Cr increased above the reference interval [2]. Therefore, serum SDMA is useful as a renal biomarker for identifying early compromise in renal function compared with serum Cr.

Symmetric dimethylarginine is produced when nitrogen molecules of arginine-containing proteins are postranslationally modified by adding methyl groups. When these proteins are subsequently degraded, free methylarginines are released into the cytosol and then enter the plasma. Symmetric dimethylarginine is eliminated primarily (≥ 90%) by renal clearance [3, 4]. Because serum SDMA is filtered by the kidneys, plasma concentrations are correlated with changes in glomerular filtration rate (GFR). The gold standard for estimating renal function is by measurement of GFR. A meta-analysis of 18 studies in humans showed that serum SDMA concentration is highly correlated with GFR [5]. We have shown that serum SDMA correlates with GFR in cats [1, 6] as well as in dogs [2].

Furthermore, serum SDMA concentrations are not affected by lean body mass in dogs [7]. A review of the literature shows that increased SDMA is caused by reduced renal function, and by itself does not contribute to progression of renal disease (reviewed in [7]). Chronic SDMA infusion in otherwise healthy mice had no effect on renal function, renal histology, blood pressure, or cardiac function even though SDMA concentrations were increased an order of magnitude and comparable to those in patients with CKD [8]. In theory, SDMA could interfere with renal function by inhibiting L-arginine uptake, yet using the gold standard GFR measurement for mice (FITC-inulin clearance) researchers could not detect even minor changes. These data strengthen the role of SDMA as a marker of renal impairment that plays no pathophysiological role in of itself.

The use of a biomarker that correlates with deceasing GFR allows dogs with CKD to be detected earlier in the disease course before serum Cr increases, and may allow earlier treatment options that delay progressive loss of kidney function, while maintaining adequate nutrition. Feeding a renal diet to dogs with IRIS stage 3 and 4 CKD is considered the current standard of care with strong evidence supporting this recommendation [9]. Dietary modifications include decreased protein, phosphorus, and sodium content; increased water soluble vitamins and fiber content; increased caloric density; and additional n-3 fatty acids (FA), antioxidants, and potassium [9]. In one study in dogs, an experimentally induced CKD remnant kidney model, feeding foods enriched in (n-3) PUFA (15%) reduced glomerular hypertension, proteinuria, tubulointerstitial fibrosis, glomerulosclerosis, and limited the production of pro-inflammatory eicosanoid mediators such as PGE_2_ and TxA_1_ [10, 11]. Studies in 6- to 8-yr-old Beagles fed dietary (n-3) FA supplements (2.5% dry matter basis) in combination with antioxidants (vitamin E, carotenoids, and lutein at concentrations comparable to those found in commercial canine renal disease foods) showed independent and additive protective effects, best explained as causing a decrease in renal oxidant injury [12]. Specifically, the rate of decline of GFR was slowed by the use of (n-3) PUFA and by the addition of dietary antioxidants [12]. In another study, obese dogs fed a high-fat food for 7 to 9 weeks, or 24 weeks, exhibited increased GFR and renal plasma flow [13].

It is unknown whether dietary interventions in nonazotemic dogs with increased serum SDMA (early renal insufficiency) will improve renal function, based on a decrease in serum SDMA concentrations. The purpose of this study was to evaluate the short term effects of a test food that contained functional lipids (fish oil), antioxidants (lipoic acid, vitamins C and E), L-carnitine, botanicals (fruits and vegetables), controlled sodium concentration, and high quality protein sources on circulating renal biomarkers and urinalysis results in healthy geriatric dogs compared with dogs fed owner’s-choice foods. The hypothesis of this study was that dogs consuming test food would show improvement in GFR based on serum renal biomarker concentrations.

## Materials and Methods

### Dogs and ethics statement

This study protocol was reviewed and approved by the Institutional Animal Care and Use Committee, Hill’s Pet Nutrition, Inc., Topeka, KS, USA (Permit Number: CP-522). Owners signed an informed consent form prior to enrollment of their dog, agreeing to comply with instructions given by their veterinarian and detailed in the consent form. Procedures were designed to avoid or minimize discomfort, distress, and pain. Dogs were monitored for signs of disease. If an adverse event occurred, the dog’s health took precedence over continuation in the feeding trial.

### Study design

This was a prospective feeding study of 6-months duration. A contract research organization recruited seventeen veterinary clinics from around the United States. Dogs were screened (physical examination, blood work, urinalysis) and selected by veterinarians from veterinary hospitals in Kansas, Oklahoma, California, New York, Pennsylvania, Missouri, Tennessee, Georgia, Colorado and Arizona. All dogs remained with their owners throughout the study. Once dogs were accepted into the study, they were randomized to receive either test food or owner’s-choice foods. Dogs were examined at baseline, and at 30, 60, 90 and 180 days. History, physical examination forms, medication records, dietary information, and both owner and veterinarian questionnaires were filled out electronically at every visit. Blood samples were obtained and submitted for analysis of selected serum analytes, and urine was collected to determine urine specific gravity (USG) and urine protein:Cr ratio, at baseline and at 3 and 6 months.

Inclusion criteria were that dogs ≤ 22.7 kg had to be ≥ 9 years old, whereas dogs > 22.7 kg had to be ≥ 7 years old at the start of the study. All dogs had to have ovariohysterectomy or be neutered. Dogs had to be free of chronic disease based on normal findings on physical examination, complete blood count, serum chemistry profile, complete urinalysis, total T4, and a negative heartworm test. Exceptions included dogs with mild arthritis, mild dermatitis, mild dental disease, or IRIS Stage 1 CKD [14]. IRIS Stage 1 CKD included dogs with serum Cr < 1.4 mg/dL plus at least one other renal abnormality including: abnormal kidneys on palpation or imaging, persistent proteinuria of renal origin, inadequate concentration of urine with no explanation other than renal origin, or concentrations of serum Cr that were progressively increasing. Dogs also had to have a prior client-patient relationship with the attending veterinarian and a history of partaking in a good preventive medicine program, including routine vaccinations, deworming, heartworm testing and prevention, and all recommended diagnostic testing performed. Up to two dogs could be enrolled from one household.

Veterinarians and owners were compensated for their participation in the study. Study food was provided free of charge to pet owners participating in the study for the duration of the study. The veterinarians and the owners were blinded as to the sponsor of the study. The owners were instructed to maintain the test food at room temperature in the original food packaging. No special instructions were provided for handling foods of owner’s-choice. If an owner asked, the response was that they should store the food as indicated according to the manufacturer’s instructions on the package.

Dogs were excluded if they were receiving long-term systemic medication or had a history of chronic disease (e.g., heart disease; endocrine disease; recurrent urinary tract disease including urolithiasis; pancreatitis; hepatic or gastrointestinal disease; severe dermatitis; and severe periodontitis). Dogs that belonged to a hospital employee of any hospital involved with the study or that weighed < 6.8 kg were not eligible. Dogs that were on a therapeutic or prescribed commercial brand pet food (other than a diet for weight loss) or that were fed raw food were not eligible. Lastly, dogs currently enrolled in another study were ineligible.

The criteria for removal of a dog from the study included: failure to consume the study food, the owner requested to leave the study, if the veterinarian felt that the dog should not continue on the study, if there was poor owner compliance (e.g., routine administration of treats, dietary supplements, table scraps, neutraceuticals, or canned food to dogs consuming the test or control foods that was in excess of 10% of daily calories), if the dog had to be changed to a different food because a disease was diagnosed necessitating a change in food, if a life-threatening illness or accident occurred to the dog or the owner and the veterinarian determined that it was best not to continue the study, if the dog died, or if the owner elected euthanasia of the dog. If an adverse event occurred, a dog could continue the study if it was able to consume test food or, if in the control group, a non-therapeutic food. Also, if an adverse event occurred, and the dog was placed on chronic medication that adequately controlled a newly diagnosed condition (e.g., nonsteroidal anti-inflammatory drug for arthritis, glucocorticoid for atopy, insulin for diabetes) and the owner and veterinarian elected not to alter the dog’s food, a dog could continue the study.

There were a total of 255 dogs enrolled in the study; mean age of 9.7 years (range 7 to 15 years). There were 117 males and 138 females. Of the dogs classified as small (6.8–11.4 kg), there were 26 fed test food and 25 fed owner’s-choice foods. Of the dogs classified as medium (11.5–22.7 kg), there were 25 fed test food and 24 fed owner’s-choice foods. Of the dogs classified as large (>22.7 kg), there were 78 fed test food and 77 fed owner’s-choice foods.

### Foods

The test food was produced by Hill’s Pet Nutrition, Inc., Topeka, KS, and met the nutritional requirements for adult dogs (≥ 1 year) as established by the Association of American Feed Control Officials (AAFCO). Food was available in dry form only. Food composition, expressed as percentage of food, as fed, is shown in **Table 1**. Test food was a renal protective food containing functional lipids (fish oil), antioxidants (lipoic acid, vitamins C and E), L-carnitine, botanicals (fruits and vegetables), controlled sodium concentration, and improved quality of protein sources (high bioavailability and an ideal amino acid composition). Test food contained 0.5% fish oil, 100 mg/kg lipoic acid, additional α-tocopherol acetate (> 1,300 IU/kg, as fed) and vitamin C (> 400 mg/kg, as fed), and 300 mg/kg L-carnitine. Test food also contained more biologically available protein sources than expected with commercially available owner’s-choice foods (egg protein and wet meat chicken replaced other protein sources). In addition, there were increased amounts of fruits and vegetables; i.e. beet pulp, citrus pulp, carrot granules, dried spinach, and tomato pomace.

**Table 1:** Food Composition of Test Food.

Nutrient^a^	Test Food
Moisture	7.77
Protein	21.75
Fat	16.48
Atwater Energy,^b^ kcal/kg	3,793
Ash	5.15
Crude Fiber	2.24
Calcium	0.91
Phosphorus	0.68
Sodium	0.18
Total tocopherols, IU/kg	1,373.04
Vitamin C, mg/kg	404.05
Lauric acid [12:0]	0.01
Myristic acid [14:0]	0.12
Palmitic acid [16:0]	3.28
Stearic acid [18:0]	0.88
LA [18:2 (n-6)]	3.32
αLA [18:3 (n-3)]	0.51
ARA [20:4 (n-6)]	0.11
EPA [20:5 (n-3)]	0.10
DPA [22:5 (n-3)]	0.02
DHA [22:6 (n-3)]	0.07
SFA^c^	4.36
MUFA^d^	6.14
PUFA^e^	4.31
(n-6) FA^f^	3.54
(n-3) FA^g^	0.72
(n-6):(n-3) ratio	4.92

^a^All analytical values are expressed as percentage of food, as fed, unless otherwise indicated.

^b^ Energy calculated using the modified Atwater factors as described [15].

^c^ Sum of the SFA: 8:0+10:0+11:0+12:0+14:0+15:0+16:0+17:0+18:0+20:0+22:0+24:0.

^d^ Sum of the MUFA: 14:1+15:1+16:1+17:1+18:1+20:1+22:1+24:1.

^e^ Sum of the PUFA: 18:2(n-6)+18:3(n-6)+18:3(n-3)+18:4(n-3)+20:2(n-6)+20:3(n-6)+20:3(n-3)+20:4(n-6)+20:4(n-3)+20:5(n-3)+21:5(n-3)+22:2(n-6)+22:4(n-6)+22:5(n-6)+22:5(n-3)+22:6(n-3).

^f^ Sum of the (n-6) fatty acids.

^g^ Sum of the (n-3) fatty acids.

Food analytical measurements and FA composition of the test food was determined by a commercial laboratory (Eurofins Scientific, Inc., Des Moines, IA) using Association of Analytical Communities (AOAC) methods. Test food FA were determined by gas chromatography of FA methyl esters. FA concentrations are expressed as g/100 g of FAs as fed. The sum of dietary SFA was determined as follows: 8:0+10:0+11:0+12:0+14:0+15:0+16:0+17:0+18:0+20:0+22:0+24:0. The sum of dietary MUFA was determined as follows: 14:1+15:1+16:1+17:1+18:1+20:1+22:1+24:1. The sum of dietary PUFA was determined as follows: 18:2(n-6)+18:3(n-6)+18:3(n-3)+18:4(n-3)+20:2(n-6)+20:3(n-6)+20:3(n-3)+20:4(n-6)+20:4(n-3)+20:5(n-3)+21:5(n-3)+22:2(n-6)+22:4(n-6)+22:5(n-6)+22:5(n-3)+22:6(n-3).

The test food was provided to the study sites by a commercial carrier, and each site received supplies of test food both before and during the study period on an as needed basis. Pet owners were instructed to store the food in the package provided according to label instructions.

The owner’s-choice foods could be any dry, non-therapeutic, non-prescription food, excluding raw foods, of the owners choosing. Owners with dogs in the control group were able to switch foods at their discretion.

Offering amounts were of equal calories based on the presumed resting energy requirement (RER) for each animal calculated by the formula: RER = 70 x (ideal body weight in kg) ^0.75^ with instructions to obtain and/or maintain ideal body weight.

### Serum and urine analyses

The same veterinary diagnostic laboratory (Marshfield Labs, Marshfield, WI) was used for determining blood hemoglobin, and serum sodium and renal function biomarker concentrations. Serum Cr and BUN concentrations were determined by enzymatic colorimetric methods. The reference intervals for blood hemoglobin (10.5 to 20.1 g/dL), serum sodium (141 to 159 mmol/L), serum Cr (0.5 to 2.0 mg/dL), and serum BUN (8.0 to 30.0 mg/dL) in adult dogs were previously established. Serum SDMA concentrations were determined using liquid chromatography-mass spectrometry as previously described [7]. The reference interval for serum SDMA in healthy dogs was < 14 μg/dL [16]. All serum SDMA concentrations were determined retrospectively after the feeding trial ended from serum stored in serum banks.

Urine specific gravity was determined using a refractometer. Urine Cr concentration was used as an internal reference and measured with the same assay as serum Cr. Urine protein concentrations were determined using urine supernatant (benzethonium chloride turbidometric method). Urine protein to creatinine (UPC) ratio calculations are reported as mg/dL protein: mg/dL creatinine.

### Statistical analyses

Statistical analyses were performed using Statistical Analysis Software version 9.2 (SAS Institute, Cary, NC). Response variables were tested for normality by the Shapiro-Wilk test. To determine the effect of food, data from dogs were analyzed as repeated-measures-in-time, randomized design using general linear models in PROC MIXED and the Satterthwaite approximation to determine the denominator degrees of freedom for the tests of fixed effects. Animal was considered the experiment unit. The variance-covariance structure of repeated measures of the same animal across time was modeled using an unstructured variance-covariance matrix, which was the most parsimonious model according to the Aikaike Information Criterion. Fixed effects in the model were treatment group (owner’s-choice foods, test food), time (0, 3, and 6 months), and their interaction. If there was a significant F-test, mean separation was completed by the PROC MIXED PDIFF statement.

In a subset of dogs that had an SDMA ≥14 μg/dL at baseline or during the 6-month feeding trial, a single measure t-test was performed to evaluate the effect of time on response variables within each treatment group. A Chi-Square test was also used to evaluate the effect of time on serum SDMA and Cr concentrations to compare treatment effects. All data are reported as least square means (LSM) ± SEM.

A correlation analysis of variables was also performed on baseline and final data to compare serum SDMA and creatinine concentrations with other response variables. Statistical significance was declared at *P* ≤ 0.05.

## Results

A total of 210 dogs completed the 6 month study. Twenty eight of 129 dogs (21.7%) consuming test food, and 17 of 126 dogs (13.5%) consuming owner’s-choice foods failed to complete the study. In the test food group, a higher drop out occurred at baseline because owners of ten dogs chose to remove their pet from the study because test food was less acceptable compared with what they had been eating. Of the 210 dogs completing the study, mean age was 9.6 years (range 7 to 15 years). There were 94 males and 116 females, with mean ± SD initial body weight of 25.0 ± 12.0 kg (**Table 2**).

**Table 2:** Dogs completing the 6 month feeding trial were classified according to age, sex, initial body weight, and whether they were fed test food or owner’s-choice (OC) foods.

	N			Age (y)				Sex				Initial Body Weight (kg)			
	Test	OC	Total	Test		Owner’s Choice		Test		Owner’s Choice		Test		Owner’s Choice	
	N	N	N	Mean	Range	Mean	Range	Male	Female	Male	Female	Mean	SD	Mean	SD
Small	21	22	43	10.19	9–14	10.82	9–13	8	13	10	12	8.67	1.54	8.52	1.21
Medium	18	22	40	10.28	9–14	11.18	9–15	6	12	8	14	16.81	2.38	18.28	3.47
Large	62	65	127	8.89	7–15	8.94	7–13	33	29	29	36	34.40	8.51	31.27	7.15
**Combined**	**101**	**109**	**210**	**9.41**	**7–15**	**9.77**	**7–15**	**47**	**54**	**47**	**62**	**25.92**	**12.95**	**24.06**	**10.96**

Concentrations of blood hemoglobin, serum sodium and renal function biomarkers, and urinalysis parameters of dogs at baseline, 3 months, and 6 months are shown in **Table 3 (**least square means ± SEM). Dogs consuming test food showed significant decreases in serum SDMA, Cr, and BUN concentrations across time. Dogs consuming owner’s-choice foods showed no significant changes in serum SDMA, Cr, and BUN concentrations across time. For serum SDMA (*P* = 0.05) and serum BUN (*P* = 0.01), there were significant interactions between diet and time on study.

**Table 3:** Hemoglobin, serum albumin, sodium, and renal function biomarkers, and urinalysis parameters of dogs at baseline (initial) and after consuming test food or owner’s-choice foods for 3 and 6 months (mean ± SEM).

	Owner’s- Choice Foods		Test Food	Two-way ANOVA Analysis^†^ (*P* values)		
Number of Animals, N	109		101	Main Effect Diet	Main Effect Time	Effect of Diet x Time
**Hemoglobin:**						
*Hemoglobin (g/dL)*				0.75	0.45	0.48
Initial	17.2 ± 0.19		17.0 ± 0.18			
3 months	16.9 ± 0.19		17.3 ± 0.18			
6 months	17.1 ± 0.19		17.0 ± 0.18			
**Albumin:**						
*Albumin (g/dL)*				0.08	0.32	0.66
Initial	3.41 ± 0.03	3.49 ± 0.03				
3 months	3.42 ± 0.03	3.44 ± 0.03				
6 months	3.46 ± 0.03	3.49 ± 0.03				
**Serum Electrolytes:**						
*Serum Sodium (mmol/L)*				0.07	0.01	0.14
Initial	147.5 ± 0.18		147.6 ± 0.18^a^			
3 months	147.1 ± 0.18		146.5 ± 0.18^b^			
6 months	147.1 ± 0.18		146.8 ± 0.19^b^			
**Renal function markers**:						
*Urea Nitrogen (mg/dL)*				<0.001	0.02	0.01
Initial	16.16 ± 0.50		16.13 ± 0.52^a^			
3 months	16.10 ± 0.50		13.55 ± 0.52^b^			
6 months	16.49 ± 0.51		13.81 ± 0.51^b^			
*Serum Cr (mg/dL)*				0.24	0.003	0.26
Initial	0.86 ± 0.02		0.92 ± 0.02^a^			
3 months	0.84 ± 0.02		0.87 ± 0.03^a^^,^^b^			
6 months	0.83 ± 0.02		0.81 ± 0.02^b^			
*Serum SDMA (*μ*g/dL)*				0.80	0.05	0.05
Initial	9.00 ± 0.22		9.66 ± 0.23^a^			
3 months	8.82 ± 0.22		8.73 ± 0.23^b^			
6 months	9.18 ± 0.22		8.76 ± 0.22^b^			
**Urinalysis parameters:**						
*Urine Specific Gravity*				0.76	0.34	0.93
Initial	1.030 ± 0.001		1.030 ± 0.001			
3 months	1.029 ± 0.001		1.029 ± 0.001			
6 months	1.029 ± 0.001		1.028 ± 0.001			
*Urine Protein*:*Cr Ratio*				0.01	0.15	0.18
Initial	0.22 ± 0.06		0.33 ± 0.06			
3 months	0.22 ± 0.06		0.36 ± 0.07			
6 months	0.30 ± 0.06		0.47 ± 0.07			

^†^*P* values are shown for diet main effects, time main effects, and for interaction of diet and time.

^a,b^Means with different superscripts within a column are different between times at *P*≤0.05.

Hemoglobin and serum albumin concentrations remained stable across time for dogs consuming both test food and owner’s-choice foods. Serum sodium concentrations decreased across time in dogs consuming test food at 3 and 6 months compared with baseline (*P* ≤ 0.05), but were stable in dogs consuming owner’s choice foods.

Urine specific gravity remained stable across time for dogs consuming both test food and owner’s-choice foods. By random allocation, dogs in the test food group started with higher urine protein:Cr ratios at baseline, and remained higher throughout the 6 month study. There was no influence of diet on change across time, and there was no interaction of diet and urine protein:Cr ratio.

The correlation between serum SDMA concentration and urine protein/creatinine ratio at baseline was not significant (for all dogs, *r* = -0.05, *P* = 0.48; for those dogs with elevated SDMA (n = 18), *r* = -0.06, *P* = 0.42). However, the correlation between serum SDMA and albumin concentrations at baseline was significant (*r* = -0.26, *P* < 0.001) and remained significant after feeding for 6 months (*r* = -0.30, *P* < 0.001). These results were similar for correlations between serum Cr and albumin concentrations at baseline and after feeding for 6 months (*r* = -0.24 and -0.21, respectively, both *P* <0.001). Serum SDMA and Cr concentrations were also unrelated to serum sodium and hemoglobin concentrations at baseline: correlations for serum SDMA and hemoglobin (*r* = -0.11, *P* = 0.13), for serum SDMA and serum sodium (*r* = -0.06, *P* = 0.40), for serum Cr and hemoglobin (*r* = 0.04, *P* = 0.57), and for serum Cr and serum sodium (*r* = 0.10, *P* = 0.14).

A subset of nonazotemic dogs with elevated serum SDMA concentrations (indicating early renal insufficiency) was also analyzed. At baseline or during the 6-month feeding trial, 18/210 dogs (8.6%) s had serum SDMA ≥14 μg/dL and serum Cr <2.0 mg/dL (**Table 4 and Table 5**). This included nine dogs fed owner’s-choice foods and nine dogs fed test food. Compared with baseline, after feeding 9 dogs owner’s-choice foods for 6 months, four improved during the course of the study (serum SDMA decreased), four got worse (serum SDMA increased), and one stayed the same (no change in serum SDMA). More dogs had decreases in serum SDMA concentrations when fed test food (*P*<0.01) compared with owner’s-choice foods. Compared with baseline, after feeding 9 dogs test food for 6 months, eight improved (serum SDMA decreased) and one got worse (serum SDMA increased). The changes in serum SDMA and Cr concentrations were significant (both *P* = 0.03) only for dogs fed test food.

**Table 4:** Hemoglobin, serum sodium and renal function biomarkers, and urinalysis parameters in a subset of dogs that had serum SDMA concentration ≥14 μg/dL (indicating renal insufficiency) at baseline or during the 6-month feeding trial.

	Owner’s-Choice Foods	Test Food
Number of Animals, N	9	9
**Hemoglobin:**^**a**^		
*Hemoglobin (g/dL)*		
Initial	16.6 ± 0.61	16.5 ± 0.58
3 months	16.4 ± 0.31	16.1 ± 0.31
6 months	16.7 ± 0.45	15.7 ± 0.45
Change	0.1 ± 0.45	-0.8 ± 0.45
P value^†^	*P* = 0.87	*P* = 0.08
**Serum electrolytes:**		
*Serum Sodium (mmol/L)*		
Initial	147.7 ± 0.71	147.4 ± 0.71
3 months	146.8 ± 0.39	146.6 ± 0.39
6 months	147.4 ± 0.59	146.7 ± 0.59
Change	-0.22 ± 0.95	-0.77 ± 0.95
P value^†^	*P* = 0.42	*P* = 0.42
**Renal function markers**:		
*Urea Nitrogen (mg/dL)*		
Initial	21.22 ± 4.41	19.55 ±1.69
3 months	21.00 ± 3.79	17.44 ± 3.20
6 months	24.11 ± 4.41	17.55 ± 2.68
Change	2.89 ± 1.54	-2.00 ± 1.78
P value^†^	*P* = 0.10	*P* = 0.30
*Serum Cr (mg/dL)*		
Initial	1.02 ± 0.08	1.22 ± 0.10
3 months	1.03 ± 0.10	1.10 ± 0.09
6 months	1.02 ± 0.09	1.01 ± 0.4
Change	0.00 ± 0.09	-0.210 ± 0.07
P value^†^	*P* = 1.00	*P* = 0.03
*Serum SDMA (*μ*g/dL)*		
Initial	12.9 ± 1.15	13.72 ± 0.75
3 months	12.5 ± 0.75	12.21 ± 0.94
6 months	13.39 ± 0.94	11.57 ± 0.83
Change	0.53 ± 1.54	-2.15 ± 0.85
P value^†^	*P* = 0.74	*P* = 0.03
**Urinalysis parameters:**		
*Urine Specific Gravity*		
Initial	1.030 ± 0.004	1.027 ± 0.003
3 months	1.029 ± 0.003	1.028 ± 0.004
6 months	1.024 ± 0.003	1.028 ± 0.003
Change	-0.006 ± 0.014	0.000 ± 0.011
P value^†^	*P* = 0.20	*P* = 0.90
*Urine Protein*:*Cr Ratio*		
Initial	0.11 ± 0.02	0.12 ± 0.04
3 months	0.13 ± 0.03	0.14 ± 0.04
6 months	0.15 ± 0.03	0.34 ± 0.22
Change	0.04 ± 0.02	0.22 ± 0.18
P value^†^	*P* = 0.09	*P* = 0.30

^a^Shown are values at baseline (initial) and after consuming test food or owner’s-choice foods for 3 and 6 months (mean ± SEM).

^†^*P* values are shown for change over time within diet.

**Table 5:** Serum SDMA and Cr biomarker concentrations, and urine specific gravity in individual dogs that had serum SDMA concentration ≥14 μg/dL (indicating renal insufficiency) at baseline or during the 6-month feeding trial.

Name, time on food	Age (y)	Sex	Serum SDMA (μg/dL)	Serum Cr (mg/dL)	Urine Specific Gravity	Overall Response^a^
**Dogs fed owner’s-choice foods**^**b**^						
*Melody*	11.5	SF				
Initial			7.1	1.0	1.036	
3 months			14.1	1.0	1.032	
6 months			11.2	1.0	1.028	Worse
*Sadie*	15	SF				
Initial			17.0	0.9	1.031	
3 months			15.9	1.0	1.028	
6 months			11.1	0.8	1.023	Improved
*Pippen*	14	NM				
Initial			12.8	0.8	1.015	
3 months			14.3	0.9	1.014	
6 months			15.7	0.9	1.016	Worse
*Lady*	13.5	SF				
Initial			11.4	0.9	1.050	
3 months			11.4	0.8	1.048	
6 months			19.6	1.4	1.010	Worse
*Misty*	14	SF				
Initial			17.8	1.4	1.037	
3 months			12.9	1.3	1.033	
6 months			13.3	1.3	1.040	Improved
*Red Dog*	15	NM				
Initial			13.8	1.4	1.026	
3 months			9.8	1.6	1.028	
6 months			13.8	1.3	1.028	Stable
*Lady*	9	SF				
Initial			8.8	0.9	1.036	
3 months			13.8	0.9	1.036	
6 months			12.7	0.7	1.031	Worse
*Tobey*	9	NM				
Initial			13.6	1.2	1.025	
3 months			10.0	1.2	1.020	
6 months			10.5	1.1	1.015	Improved
*Casper*	14	NM				
Initial			13.5	0.7	1.015	
3 months			9.9	0.6	1.024	
6 months			12.6	0.7	1.022	Improved
**Dogs fed test food**:						
*Koby*	13.5	NM				
Initial			14.2	1.3	1.024	
3 months			15.0	1.1	1.027	
6 months			14.0	1.1	1.032	Improved
*Griffin*	15	NM				
Initial			14.7	1.7	1.027	
3 months			14.7	1.6	1.035	
6 months			12.0	1.1	1.038	Improved
*Jasmine*	9	SF				
Initial			14.1	1.4	1.051	
3 months			10.8	1.2	1.042	
6 months			10.2	1.1	1.037	Improved
*Zeus*	8	NM				
Initial			14.6	1.1	1.031	
3 months			7.1	1.0	1.022	
6 months			7.5	0.9	1.020	Improved
*Buffy*	10	SF				
Initial			15.7	1.4	1.016	
3 months			10.4	1.3	1.037	
6 months			13.7	1.2	1.028	Improved
*Casey*	13.5	NM				
Initial			15.4	1.5	1.030	
3 months			13.7	1.2	1.018	
6 months			12.6	1.0	1.041	Improved
*Samson*	13.5	NM				
Initial			8.7	0.8	1.022	
3 months			14.8	0.7	1.012	
6 months			7.7	0.9	1.015	Improved
*Yensen*	9.5	SF				
Initial			14.7	0.9	1.027	
3 months			NA	0.9	1.018	
6 months			13.0	1.0	1.030	Improved
*Bella*	8.5	SF				
Initial			11.4	0.9	1.019	
3 months			11.1	0.9	1.041	
6 months			13.5	0.8	1.010	Worse

^a^Based on change in serum SDMA concentration over the 6-month feeding period.

^b^Shown are values at baseline (initial) and after consuming owner’s-choice or test foods for 3 and 6 months.

## Discussion

Glomerular filtration rate is directly related to functional renal mass. We have previously shown in dogs that serum SDMA concentrations are inversely related to GFR [2], that serum SDMA concentrations can be used to detect renal dysfunction earlier in dogs with chronic renal disease compared with serum Cr concentrations [2], and that serum SDMA concentrations are not affected by lean body mass [7]. The upper reference interval for SDMA (< 14 μg/dL) corresponds to a reduction in GFR of approximately 49% from mean GFR, whereas the upper reference interval for serum Cr corresponds to a reduction of approximately 75% from mean GFR in dogs [2]. On average, serum SDMA detects a reduction in GFR 9.8 months before serum Cr in dogs with chronic renal disease [2].

The purpose of this study was to determine if dietary interventions in nonazotemic dogs with increased serum SDMA concentrations (early renal insufficiency) could improve renal function based on a decrease in serum SDMA. Our results suggest that dogs with early renal insufficiency fed a test food designed to promote healthy aging, over a 6 month period, were more likely to have improved renal function evidenced by a decrease in or stable serum SDMA concentrations compared with dogs fed owner’s-choice foods.

Because abnormal serum sodium concentrations are common in human CKD patients, both hypo- and hypernatremia, their risk increases with advancing stage of CKD, and they are associated with a significant increase in mortality [17], we also assessed serum sodium concentrations in dogs overall, as well as in the subset of nonazotemic dogs with increased serum SDMA concentrations (indicating early renal insufficiency). Serum sodium concentrations decreased across time in dogs consuming test food, but were stable in dogs consuming owner’s- choice foods. Changes may be biologically insignificant as concentrations remained within the reference interval. Also, these changes were not unexpected because sodium concentration of the test food was controlled. Hemoglobin concentrations also remained stable across time for dogs consuming both test food and owner’s-choice foods.

We found no significant relationship between serum SDMA concentrations and urine protein/creatinine ratios at baseline. However, it was of interest in this population that both serum SDMA and creatinine concentrations at baseline and after feeding for six months were negatively associated with serum albumin concentrations.

Test food was energy-dense and contained functional lipids (fish oil), antioxidants (lipoic acid, vitamins C and E), L-carnitine, botanicals (fruits and vegetables), highly bioavailable protein (egg and wet meat chicken), and increased amino acids (ideal amino acid composition). Traditional nutritional studies have focused on individual nutrients or foods, but their additive or interactive influences are more apparent when complete diets or several nutritional interventions in combination are studied in healthy aging trials [18]. In humans, the decline in renal function that occurs in a large percentage of aging and CKD populations is likely linked to increased levels of oxidative stress and inflammation [19]. Food is a major source of oxidants, and diets can be modified to decrease oxidant burden [19]. In this study, feeding test food for 6 months reversed the increase in serum SDMA concentration in seven of nine dogs, and serum SDMA concentration remained stable in one dog.

Observational studies in humans consuming diets rich in fruits and vegetables demonstrated a reduced risk of incident CKD by increasing the dietary alkali load, thereby reducing net endogenous acid production as reviewed in [20]. A proinflammatory diet, based on the assumed proinflammatory effects of certain nutrients, vitamins and trace elements, is associated with systemic inflammation as well as with reduced kidney function [21]. Thus, inflammation may be one of the pathways through which diet can affect kidney function [21].

Test food contained higher amounts of (n-3) FA than most commercially available foods. Dietary PUFA, including EPA and DHA, influence the physical nature of cell membranes and membrane protein-mediated responses, lipid-mediator generation, cell signaling, and gene expression in many different cell types [22]. The (n-3) PUFA from fish oil, and lysophospholipids [23] and eicosanoids derived from EPA and DHA may protect against excessive inflammatory reactions. Previous studies in dogs with a remnant kidney model of CKD have shown that feeding foods enriched in (n-3) PUFA (15%) attenuates glomerular hypertension, proteinuria, tubulointerstitial fibrosis, glomerulosclerosis, and limits the production of pro-inflammatory eicosanoid mediators such as PGE_2_ and TxA_1_ [10, 11].

In unsupplemented human CKD patients, renal dysfunction is associated with decreased plasma vitamin C levels, which may cause endothelial dysfunction via an increase in oxidative stress [24]. A large-scale study of an elderly population reported an increased risk for all-cause mortality with decreased plasma vitamin C levels [25]. Vitamin E levels were higher in healthy subjects compared with subjects in all other stages of renal function decline [26]. Oral supplementation of vitamins C and E, in combination [27, 28], or as a micronutrient cocktail containing physiologic doses of antioxidant vitamins and trace minerals [28], can decrease oxidative stress in humans. Using rats, others have shown that vitamin E ameliorates the decline in GFR associated with aging and oxidative stress [29], and that co-supplementation of vitamins E and C prevents gentamicin-induced nephrotoxicity and preserves GFR [30]. In a pig experimental model of renovascular disease (renal artery stenosis), chronic blockade of the oxidative stress pathway with vitamins C and E improved renal blood flow, GFR, regional perfusion responses to challenge, and blunted inflammation and fibrosis in the stenotic kidney [31]. In humans, oxidative stress is common in CKD patients and is considered to be an important pathogenic mechanism [32]. The majority of studies investigating anti-oxidant treatments in CKD patients show a reduction in oxidative stress and many show improved renal function (reviewed in [32, 33]). Thus, diet may affect kidney function by altering the balance between antioxidants and oxidizing species.

Dietary supplementation with L-carnitine is associated with improved nitrogen balance, inhibition of apoptosis, improved mitochondrial function, and antioxidant and anti-inflammatory effects [34]. L-carnitine is needed to transport long-chain FA from the cytosol to sites of FA β-oxidation in the mitochondria. Its content decreases with age in rats [35, 36] and dogs [37]. Acetate is the end product of FA β-oxidation and provides usable energy via the citric acid cycle. Odd-chain FA and butyrate are synthesized primarily by the intestinal microbiome, whereas other short-chain FA are derived from endogenous catabolism of branched-chain AA and lysine. Under normal conditions, the availability of carnitine is not a limiting step in FA utilization, although an increase in carnitine content may lead to glucose and AA sparing, preserving muscle glycogen content, and ensuring maximal rates of oxidative ATP production [38].

L-carnitine supplementation also has been shown to decrease markers of oxidative stress and inflammation in patients with chronic diseases such as CKD as reviewed in [34]. For example, evidence shows that carnitine prevents oxidative stress and inflammation by inhibiting production of reactive oxygen species and inflammatory cytokines. A recent meta-analysis supports a clinical benefit of L-carnitine supplementation in lowering circulating levels of C-reactive protein [39]. We previously showed that serum concentrations of carnitine and fatty acyl carnitines were decreased in geriatric dogs (>7 years), and that dietary supplementation with L-carnitine attenuated the age-associated decline in circulating carnitine concentrations [37]. The effects of supplementing L-carnitine on GFR are unknown, but in rats nephrotoxicity and oxidative stress induced by doxorubicin showed that L-carnitine administered 1 hour before doxorubicin injection and daily thereafter for 15 d increased GRF and effective renal plasma flow [40].

In conclusion, 8.6% of client-owned, geriatric dogs with early stage kidney disease, consistent with IRIS Stage 1 CKD, were identified with increased serum SDMA and normal serum Cr concentrations over a 6-month period. Those dogs that were switched to a test food that contained functional lipids (fish oil), antioxidants (lipoic acid, vitamins C and E), L-carnitine, botanicals (fruits and vegetables), highly bioavailable protein (egg and wet meat chicken), and increased amino acids (ideal amino acid composition) were more likely to reverse the increase in serum SDMA than dogs that continued to consume foods of owner’s-choice. These results suggest that nonazotemic dogs with increased serum SDMA fed a food designed to promote healthy aging are more likely to demonstrate improved renal function compared with dogs fed owner’s-choice foods.
